# Expanding the Evidence of a Semi-Dominant Inheritance in *GDF2* Associated with Pulmonary Arterial Hypertension

**DOI:** 10.3390/cells10113178

**Published:** 2021-11-15

**Authors:** Natalia Gallego, Alejandro Cruz-Utrilla, Inmaculada Guillén, Amparo Moya Bonora, Nuria Ochoa, Pedro Arias, Pablo Lapunzina, Pilar Escribano-Subias, Julián Nevado, Jair Tenorio-Castaño

**Affiliations:** 1 Instituto de Genética Médica y Molecular (INGEMM), IdiPaz, Hospital Universitario La Paz, 28046 Madrid, Spain; natalia.gallego.zazo@idipaz.es (N.G.); palajara@gmail.com (P.A.); plapunzina@gmail.com (P.L.); jnevadobl@gmail.com (J.N.); 2 CIBERER, Centro de Investigación en Red de Enfermedades Raras, Instituto de Salud Carlos III, 28029 Madrid, Spain; 3 ITHACA, European Reference Network on Rare Congenital Malformations and Rare Intellectual Disability, 75019 Paris, France; 4 Pulmonary Hypertension Unit, Department of Cardiology, Hospital Universitario 12 de Octubre, 28041 Madrid, Spain; acruzutrilla@gmail.com (A.C.-U.); nuriaochoaparra@hotmail.com (N.O.); pilar.escribano.subias@gmail.com (P.E.-S.); 5 Centro de Investigación Biomédica en Red en Enfermedades Cardiovasculares, Instituto de Salud Carlos III (CIBERCV), 28029 Madrid, Spain; 6 Pediatric Cardiology Unit, Department of Pediatrics, Hospital Universitario Virgen del Rocío, 41013 Sevilla, Spain; miguillenr@hotmail.com; 7 Pediatric Cardiology Unit, Department of Pediatrics, Hospital Universitario La Fe, 46026 Valencia, Spain; amparmoya@gmail.com; 8 ERN, European Reference Network Pulmonary Hypertension, 60590 Frankfurt, Germany

**Keywords:** *GDF2*, hereditary hemorrhagic telangiectasia, pulmonary arterial hypertension, massive parallel sequencing, NGS, genomic medicine, personalized medicine

## Abstract

Pulmonary arterial hypertension (PAH) sometimes co-exists with hereditary hemorrhagic telangiectasia (HHT). Despite being clinically diagnosable according to Curaçao criteria, HHT can be difficult to diagnose due to its clinically heterogenicity and highly overlapping with PAH. Genetic analysis of the associated genes *ACVRL1*, *ENG*, *SMAD4* and *GDF2* can help to confirm or discard the presumptive diagnosis. As part of the clinical routine and to establish a genetic diagnosis, we have analyzed a cohort of patients with PAH and overlapping HHT features through a customized Next Generation Sequencing (NGS) panel of 21 genes, designed and validated in-house. We detected a homozygous missense variant in *GDF2* in a pediatric patient diagnosed with PAH associated with HHT and a missense variant along with a heterozygous deletion in another idiopathic PAH patient (compound heterozygous inheritance). In order to establish variant segregation, we analyzed all available family members. In both cases, parents were carriers for the variants, but neither was affected. Our results expand the clinical spectrum and the inheritance pattern associated with *GDF2* pathogenic variants suggesting incomplete penetrance and/or variability of expressivity with a semi-dominant pattern of inheritance.

## 1. Introduction

Pulmonary arterial hypertension (PAH) is a severe disease defined as a persistently elevated mean pulmonary artery pressure (mPAP ≥ 20 mmHg) with a mean pulmonary arterial wedge pressure (PAWP) ≤ 15 mmHg and pulmonary vascular resistance (PVR) ≥ 3 UW [1]. PAH causes progressive right-sided heart failure leading to premature death if it remains untreated [1,2]. It is known that there is a genetic component that contributes to a predisposition to develop the disease. Pathogenic variants in the gene encoding the bone morphogenetic protein type 2 receptor (*BMPR2*), a receptor for the transforming growth factor-beta (TGF-β) superfamily, explain approximately 20–25% of idiopathic PAH (IPAH) cases and 60% of heritable PAH (HPAH) [3,4,5]. 

The etiology of PAH is highly heterogeneous, and it occasionally coexists with distinct clinical entities such as hereditary hemorrhagic telangiectasia (HHT). HHT is characterized by the presence of epistaxis, mucocutaneous telangiectasias and multiple arteriovenous malformations (AVMs) in the lung, gastrointestinal tract, liver, brain, nasal mucosa, spine, and conjunctiva [6]. The AVMs consist of the lack of intervening capillaries and result in direct connections between arteries and veins [6]. According to the Curaçao criteria [7,8], the diagnosis of HHT is established in a patient with at least three of the following clinical features: epistaxis, mucocutaneous telangiectases, visceral AVMs, and/or a family history of HHT. HHT might be sometimes difficult to be diagnosed because observable manifestations may be absent until adulthood [9]. Therefore, genetic testing is considered the gold standard in the pediatric population for patients with a family history of HHT and even when genetic test results are positive, it will confirm the diagnosis regardless of family history or symptoms.

Currently, all known genetic defects that cause HHT are found within the TGF-β signaling pathway. Identification of pathogenic variants in activin A receptor type I-like (*ACVRL1* or *ALK1*), endoglin (*ENG*), growth differentiation factor 2 (*GDF2*, also known as *BMP9* or *SMAD9*), or SMAD family member 4 (*SMAD4*) establish a definitely molecular diagnosis, even if the clinical features are inconclusive. On endothelial cells, BMPR2 and ACVRL1 participate in a complex signaling in which ENG acts as a co-receptor and SMAD1, SMAD4 and SMAD9 as signaling intermediaries [10,11,12,13]. These results provide further evidence of the importance of BMP signaling and its dysregulation in the pathogenesis of PAH and HHT. Pathogenic variants in *ENG* and *ACVRL1*, count 80% of the total number of patients with HHT [14,15,16,17]. Also, pathogenic variants in *SMAD4* are responsible for a combined syndrome of juvenile polyposis with or without HHT [MIM# 174900, 175050], which occurs approximately in less than 2% of the HHT patients [18]. Finally, pathogenic variants in *GDF2* have been described in PAH patients [19,20,21] and were shown to cause an HHT-like phenotype [22].

Genetic analysis of the associated genes is extremely useful to confirm the initial presumptive diagnosis, differential diagnosis with PAH, progression and management of the disease and family risks based on the results. In families where there is an affected member with a known disease-causing variant and for those family members who do not manifest symptoms of HHT, diagnostic genetic testing is a more cost-effective solution than conventional clinical screening and may benefit from early treatment, if necessary [23]. Therefore, in order to address the molecular diagnosis of PAH patients, we applied a custom NGS panel, which included genes *ACVRL1*, *ENG*, *SMAD4* and *GDF2* and 17 additional genes related to PAH [24]. In the present study, we have identified an homozygous missense pathogenic variant in *GDF2* in a patient with PAH-HHT and a missense variant and a copy number variant (CNV) in a patient with IPAH. In both cases, their healthy parents were carriers of the same *GDF2* variants in heterozygosis state. These results add more evidence of the semi-dominant inheritance with variable expression and incomplete penetrance model related to *GDF2* variants. This also supports the co-exist variable clinical spectrum of PAH and HHT in individuals with *GDF2* pathogenic variants. 

## 2. Materials and Methods

This study was approved by the ethical committee for scientific research of each participant center and by the ethical committee of the La Paz University Hospital (CEIC-HULP PI-1210). All patients or parents of the children involved in this project signed a clinical consent form to accept their participation. 

### 2.1. Cases Presentation

The index patients included in the study were selected from the Spanish pediatric PAH registry (REHIPED). Prior to genetic diagnosis, family history information was collected. DNA samples from the proband and parents were extracted. Both probands and first-degree relatives were enrolled in the study after informed written consent was obtained in each case. The most relevant clinical information of the two included patients is summarized in Table 1.

Patient 1 was evaluated for the first time in 2013, when he was five years old. He complained of shortness of breath on exertion and recurrent epistaxis. Peripheral oxygen saturation was normal at that time. A transthoracic echocardiography (TTE) revealed indirect data of significant pulmonary hypertension. During the first right heart catheterization (RHC), mean pulmonary artery pressure (mPAP) was 55 mmHg, pulmonary artery wedge pressure (PAWP) was 8 mmHg, and pulmonary vascular resistance (PVR) was 5.4 wood units (WU). The cardiac index (CI) was surprisingly high (8.7 L/min/m^2^), consistent with the initial diagnosis of PAH related with high cardiac output. Nevertheless, neither of the parents presented symptoms suggestive of the disease and there was no known relevant family history. Despite the patient being initially diagnosed with IPAH, further examinations revealed the presence of telangiectasias on the face and back, which led to the diagnosis being extended to “possible PAH associated with HHT” according to the Curaçao criteria [7,8]. Although fecal occult blood tests showed positive results, neither a computed tomography nor a colonoscopy demonstrated the presence of arteriovenous malformations in the gastrointestinal tract. Double initial oral therapy with tadalafil (40 mg/24 h) and bosentan (125 mg/12 h) was initiated from the diagnosis, maintaining the patient stable to date. 

Patient 2 was also diagnosed in 2013 in the setting of chest pain evaluation when she was four years old. Similarly, the TTE showed data of significant pulmonary hypertension. Then, the RHC demonstrated a mPAP of 33 mmHg, a PAWP of 6 mmHg, a CI of 4.2 L/min/m^2^ and PVR of 6.5 WU. In this patient, neither the index case nor their first-degree relatives had any relevant signs or symptoms suggestive of HHT. At diagnosis, thoracic computed tomography was normal. Equally, blood samples had always been normal, without evidence of anemia during follow-up. Oxygen saturation at rest and during exercise was likewise unremarkable. Currently, the patient remains stable without any limitation in her daily life, estimating a functional class I at her twelve years of age, under double oral therapy with macitentan and tadalafil. 

### 2.2. Next Generation Sequencing

Genetic analysis was performed in the probands and on their parents after obtaining DNA from peripheral blood. Samples were included into a customized Next Generation Sequencing (NGS) panel of 21 genes (HAP v1.2) designed in-house, which included: *ACVRL1*; *GDF2*; *BMPR1B*; *BMPR2*; *CAV1*; *EIF2AK4*; *ENG*; *KCNA5*; *KCNK3*; *NOTCH3*; *SMAD1*; *SMAD4*; *SMAD5*; *SMAD9*; *TBX4*; *TOPBP1*; *SARS2*; *CPS1*; *ABCC8*; *CBLN2*; *MMACHC*. This custom panel was designed using NimbleDesign tool (Roche, Indianapolis, IN, USA). Fragmentation and library preparation were performed with SeqCap EZ Choice Enrichment Kit (Roche, Indianapolis, IN, USA). Sequencing was performed with the Illumina MiSeq platform (Illumina, San Diego, CA, USA) following the manufacturer’s instructions [25].

**Figure 1:**
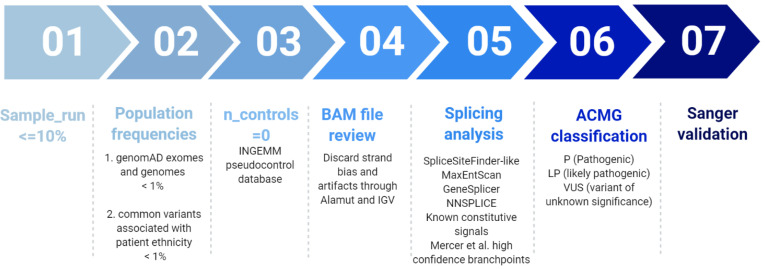
Filtering pipeline for NGS panel HAP v1.2. Modified from Tenorio et al. 2020 [25].

Variant prioritization was performed according to a custom filtering algorithm considering quality of the base pair reads, variant allele frequency in pseudo control populations and pathogenicity predictors as previously described [25], and summarized in Figure 1. Finally, variants were classified according to the guidelines described by the American College of Medical Genetics and Genomics (ACMG) [26]. 

Additionally, in order to carry out the analysis of CNVs, we used a custom script developed in-house called “LACONv” v1 (https://github.com/kibanez/LACONv, accessed on 21 June 2021) [27].

### 2.3. Segregation Analysis

In addition to the study of the probands, the analysis of all available first-degree relatives of the index cases was completed. Samples were obtained from the parents of the proband and siblings and the genetic segregation study was performed by sanger sequencing.

In patient 2, we performed a SNP array to confirm the deletion detected in the proband and her relatives. For this purpose, a SNP array study was carried out using the Infinium OmniExpressExome 8 v1.6 platform (Illumina). Image data were analyzed using the Chromosome Viewer tool contained in the Genome Studio package (Illumina, San Diego, CA, USA). In Chromosome Viewer, gene call scores < 0.15 at any locus were considered “no calls”. In addition, allele frequency analysis was applied for all SNPs. All genomic coodinates were established according to the 2009 human genome build 19 (GRCh37/NCBI build 37.1). Deletion sizes were plotted on the genome browser using the University os California at Santa Cruz Genome Browser (http://genome.ucsc.edu/, accessed on 25 June 2021).

In unaffected carriers, a complete and specific diagnostic follow-up was performed in order to rule out the disease.

## 3. Results

**Figure 2:**
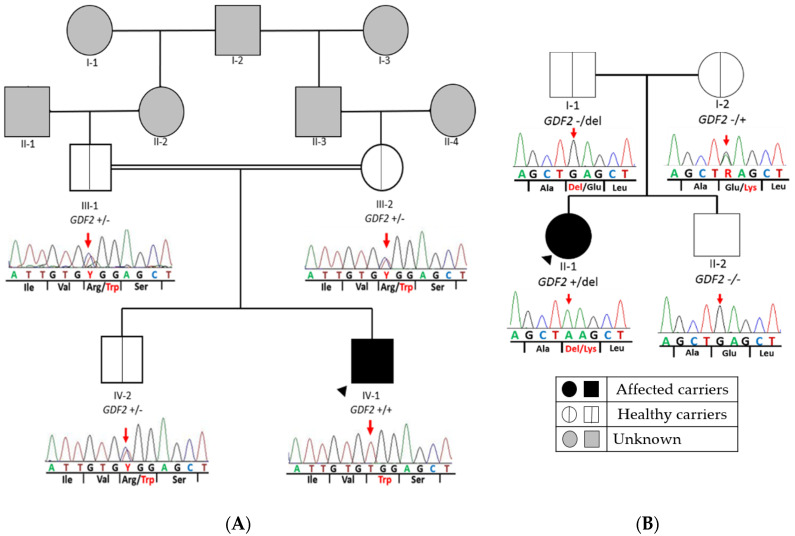
Pedigree of both families with *GDF2* variants. Segregation analysis of *GDF2* in all available family members. The figure shows a fragment of the *GDF2* sequence with corresponding amino acids below. (**A**) Patient 1: It represents the homozygous missense variant at nucleotide position c.328 (c.328C > T) in exon 1 of *GDF2* (NM_016204.3), which causes the amino acid substitution arginine to tryptophane at peptide position 110 (p.Arg110Trp) in the index patient. In addition, it shows the same heterozygous missense variant found in healthy parents and brother. (**B**) Patient 2: The missense variant at nucleotide position c.445 in exon 2 of *GDF2* (NM_ 016204.4), which causes the amino acid substitution glutamine to lysine at peptide position 149 in the index patient and mother and the deletion inherited from her father. Double line denotes consanguinity. Legend: +/+ homozygous for the alternative allele; +/− heterozygous for the alternative allele; del: deletion.

In patient 1, we identified a homozygous missense variant in exon 1 of *GDF2* (Table 2) in the index patient (*GDF2*:NM_016204.4:c.328C > T:p.(Arg110Trp)). This variant causes a substitution of an arginine to tryptophan at amino acid position 110. The parents and brother were heterozygotes for the same variant, suggesting that the child inherited a copy from each parent (Figure 2A). In silico analyses showed that this variant was absent in several population databases, and the majority of the pathogenic prediction tools (dbNSFP) suggested a damaging effect for this variant. Therefore, this variant was classified as likely pathogenic variant (LP) by applying the ACMG criteria (Table 2). 

**Figure 3:**
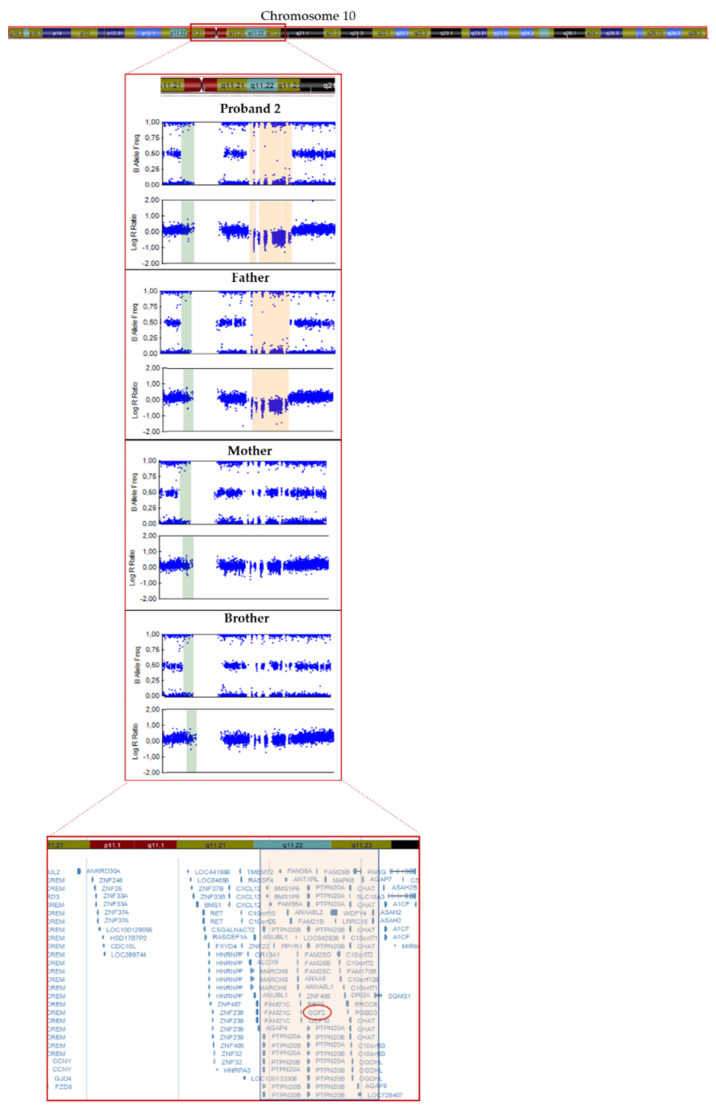
Resolution of the SNP array carried out in proband 2 and family members showing the 4.2 Mb deletion in q11.22−q11.23 region of chromosome 10 (in orange).

In patient 2, we detected a missense variant (*GDF2*:NM_016204.4:c.445G > A:p.(Glu149Lys)) in exon 2 of *GDF2* (Table 2), absent in pseudo control population databases. Based on the results of the variant segregation analysis, we determine that this variant has been inherited from her mother. This patient also inherited a deletion from her father of a size of 4.24 Mb located on chromosome 10, in the region q11.22–q11.23, which includes 41 genes (*AGAP4*, *FAM25E*, *PTPN20B*, *SYT15*, *GPRIN2*, *ANXA8L1*, *FAM25G*, *NPYAR*, *FAM25BP*, *AGAP10P*, *ANTXRL*, *ASAH2B*, *AGAP9*, *ANXA8*, *ZNF488*, *RBP3*, *GDF2*, *GDF10*, *FAM25C*, *FRMPD2*, *MAPK8*, *ARHGAP22*, *WDFYA*, *LRRC18*, *VSTM4*, *C10orf128*, *DRGX*, *ERCC6*, *CHAT*, *SLC18A3*, *OGDHL*, *PARG*, *AGAP7*, *TIM23*, *NOCOA4*, *MSMB*, *AGAP6*, *ASAH2*), which included *GDF2* (Figure 3). 

## 4. Discussion

The recent advances in massive parallel sequencing have contributed to an improvement in the knowledge of the genetic basis of PAH. Although during many years it was thought that only the primary forms of PAH were associated with a genetic factor, several studies have demonstrated that associated forms such as PAH associated with HHT also had a genetic contribution [22]. As far as we know, HHT has been classically described as an autosomal-dominantly inherited vascular malformation syndrome since the detection of heterozygous variants in *ACVRL1*, *ENG*, *SMAD4* and *GDF2* [14,15,16,17]. 

Variants in *GDF2* have been the most recent genetic discover related to PAH associated with HHT [22] and it is one of the genes with higher level of evidence of association with the development of PAH [28]. According to the analyses of the variant proteins performed by Wooderchak et al. (2013), pathogenic variants in *GDF2* negatively affect protein processing and/or function; a bmp9-deficient zebrafish model demonstrated that BMP9, encoded by *GDF2*, is involved in angiogenesis [22]. These data confirm a genetic contribution of a vascular-anomaly syndrome that has phenotypic overlap with HHT [22]. This is directly related to the main biological processes dysregulated in PAH: proliferation, apoptosis and endothelial cell migration.

In the present study, we identified two missense variants in *GDF2* and a CNV, which includes this gene in a PAH-HHT patient but also in an idiopathic PAH case. In patient 1, segregation analysis of the variant has allowed to confirm that both parents and older brother were heterozygous carriers of the same variant in *GDF2*. There is increasing evidence of homozygous variants in *GDF2* associated with HHT. In 2016, Wang et al., detected a homozygous nonsense pathogenic variant in a Chinese boy with a suggestive diagnosis of HHT but the consanguineous parents were heterozygous and apparently unaffected carriers. However, it must be noted that the father and his paternal uncle had had epistaxis meanwhile the mother and his younger sister showed vascular lesions. Sanger sequencing revealed the same homozygous variant in his sister, but at the time of publication she did not show any symptoms of PAH [29].

As in the previous case, our consanguineous parents of the patient and brother were heterozygous carriers of the same *GDF2* variant but they did not present any symptoms at the time of this study and there is no known family history. In addition, both consanguineous parents carry the same heterozygous variant, so we speculate that the variant was inherited in heterozygous form from the shared grandfather, through any of their parents. In other words, we speculate that there are at least three additional family members (I-2, II-2 and II-3 in Figure 2A) with the same heterozygous variant who had not developed the disease. Hypothetically, these results support the idea of an incomplete penetrance or variable expressivity may occur in heterozygous *GDF2* carriers. Moreover, the older brother (IV-2 in Figure 2A), who is 16 years old, presented the same heterozygous variant, but he did not show any symptoms. This may be explained by the fact that epistaxis caused by telangiectasias in the nasal mucosa eventually develop in 95% of cases but only 50% of individuals report having nosebleeds before the age of 10 years [9] and, similarly, oral or dermal telangiectasias are not usually observed until the third decade of life [22,30]. As we have already commented, in many cases an early diagnosis of the disease is difficult, thus demonstrating the great importance of genetic screening.

As in several patients previously described, our patient presented with clinical features of *GDF2*-like disorder including epistaxis and dermal lesions described as telangiectases but whose location and appearance are atypical compared to HHT patients. Meanwhile patients with homozygous variants in *GDF2* showed spider-like/linear telangiectases only on their chin and/or cheeks; patients with variants in *ACVRL1*, *ENG* and *SMAD4* have them typically on the lips and oral cavity [22]. 

Not only have homozygous variants in *GDF2* been detected in patients with HHT, but they have also been identified in patients with PAH [20,21,31,32]. Previous studies have measured BMP9 and pBMP10 plasma levels along with the serum-derived endothelial BMP activity in patients with homozygous nonsense *GDF2* mutations, PAH patient and a patient with “HHT-like” phenotype. They confirmed that it results in reduced plasma BMP9 and pBMP10 levels, even in asymptomatic heterozygous parents [33]. Furthermore, the variant specifically detected in patient 1 in this study has been previously described in heterozygosis by Hodson et al. [32] in a female patient diagnosed at 46 years with PAH. She manifested a functional class 2 of the World Health Organization (WHO) scale, with a mPAP of 38 mmHg, PAWP 5 mmHg, PVR of 6.9 WU and a CO 4.8 L/min, with no family history [32]. Hodson et al. confirmed that this variant exhibited impaired BMP9 processing, secretion or stability, thus indicating that this variant is loss-of-function and it is likely the cause of PAH. Loss of function of BMP9 may reduce the expression of BMPR2 and ALK-1 and disrupt its interaction with ENG, both of which may contribute to PAH pathogenesis [32]. 

Likewise, in patient 2, we detected a missense variant of unknown significance inherited from the mother and a CNV, which includes *GDF2* inherited from her father. The missense variant is absent in pseudo control population databases and has not been previously described. Although both parents were heterozygous for those variants, neither of them is affected. Strikingly, this proband is an idiopathic patient and she does not meet any of the Curaçao criteria. Likewise, although the CNV includes 40 genes more, the individual did not have reported comorbidities. Previous studies have reported other patients with similar deletions (4.29 Mb and 4.28 Mb) who exhibited earlier onset of disease and with no family history [32]. However, to the best of our knowledge, no segregation analysis of the variants was carried out. In view of all the above we suggest that the presence of one of the variants is not enough for the development of the disease but that the presence of a second hit is necessary. However, it would be necessary to extend this study with functional studies.

The analysis through our customized gene panel within the diagnostic routine has allowed us to describe a homozygous variant in *GDF2* and a compound heterozygous of *GDF2*. Therefore, the design of our customized panel has proven to be effective for genetic diagnosis of conflicting cases of PAH and HHT or even with overlapping features. Moreover, it was possible to detect heterozygous variants in healthy relatives, which will help them to receive detailed follow-up of their progression with the aim of early diagnosis and/or prevention of the disease. Our work adds evidence to the notion that variants in the *GDF2* may generate a greater predisposition to develop both idiopathic PAH and a “HHT-like” syndrome. Likewise, we described five healthy individuals with heterozygous variants, suggesting that there is a wider spectrum of inheritance patterns associated with *GDF2* variants, an incomplete penetrance or even the need for the presence of a second mutational event in an individual for the development of the disease.

Finally, in view of the results obtained, the importance of genetic counselling for families is clear, which can be useful when planning future pregnancies or if there is family history of a condition.

## Figures and Tables

**Table 1:** Clinical information. Main clinical characteristics of the patients and results obtained in the tests performed from diagnosis to the present time.

Number Patient	Patient 1	Patient 2
Etiology	Group 1: PAH associated with HHT	Group 1: idiopathic PAH
Sex	Male	Female
Age at diagnosis (years)	5	4
Current age (years)	13	12
NYHA functional class	II	I
Last known PAH therapies	Bosentan: 125 mg BID Tadalafil: 40 mg/day	Macitentan: 10 mg/day Tadalafil: 40 mg/day
Cardiac catheterization (mPAP and cardiac index)	5 y.o. ^1^: 55 mmHg; 8.7 L/min/m^2^ 8 y.o.: 20 mmHg	4 y.o.: 33 mmHg; 4.2 L/min/m^2^ 9 y.o.: 44 mmHg; 3.6 L/min/m^2^
Abdominal and Thoracic computed tomography	5 y.o.: No radiologic abnormalities of significance	4 y.o.: No radiologic abnormalities of significance
Cranial nuclear magnetic resonance	6 y.o.: No significant abnormalities 11 y.o.: No significant abnormalities	Not performed
Gastrointestinal endoscopy	8 y.o.: No telangiectasias detected	Not performed

^1^ y.o.: years old.

**Table 2:** Variants information.

Gene Name	Chr. Coordinate	cDNA Position ^1^	Protein Position	Variant Effect	ACMG Classification	Population Frequency ^4^
*GDF2*	Chr10:48416366	c.328C > T	p.(Arg110Trp)	Missense	LP ^2^	Absent
*GDF2*	Chr10:48414423	c.445G > A	p.(Glu149Lys)	Missense	VUS ^3^	Absent

^1^ The version of the reference genome used is hg19. The transcript used for variant annotation is: *GDF2* (NM_016204.4); ^2^ VUS: Variant of unknown significance. ^3^ LP: likely pathogenic. ^4^ Population frequency was obtained from gnomAD genomes (European non-Finnish).
